# Like a shot‐through manubrium: A rare presentation of skeletal tuberculosis

**DOI:** 10.1002/ccr3.7119

**Published:** 2023-05-01

**Authors:** Tomohiro Fujiwara, Hiroyuki Yanai, Hideharu Hagiya

**Affiliations:** ^1^ Department of Orthopaedic Surgery Okayama University Graduate School of Medicine, Dentistry and Pharmaceutical Sciences Okayama 700‐8558 Japan; ^2^ Department of Diagnostic Pathology Okayama University Graduate School of Medicine, Dentistry and Pharmaceutical Sciences Okayama 700‐8558 Japan; ^3^ Department of General Medicine Okayama University Graduate School of Medicine, Dentistry and Pharmaceutical Sciences Okayama 700‐8558 Japan

**Keywords:** manubrium, sternal infection, tuberculosis

## Abstract

A 22‐year‐old Vietnamese woman presented with anterior chest swelling. Computed tomography revealed an osteolytic lesion in the manubrium, whereas MRI showed an extra‐osseous expansion. A needle biopsy showed granuloma formation, whereas a 3‐week mycobacterial culture indicated *Mycobacterium tuberculosis* infection. Manubrium/sternum involvement in tuberculosis is extremely rare but should be considered.

## 1 CASE PRESENTATION

**FIGURE 1:**
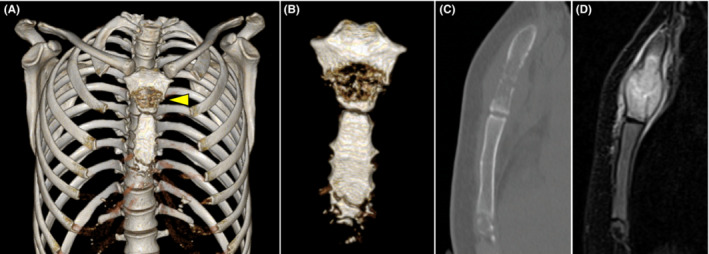
Computed tomography (CT) and magnetic resonance (MR) images of the diseased manubrium/sternum. (A, B) Three‐dimensional reconstruction of the CT image; arrowheads indicate osteolysis of the manubrium. (C) Sagittal CT image; arrowheads indicate osteolysis of the manubrium. (D) Sagittal T2‐weighted MR image; arrowheads indicate the high‐signal intensity in the bone marrow of the manubrium and the extra‐osseous expansion.

**FIGURE 2:**
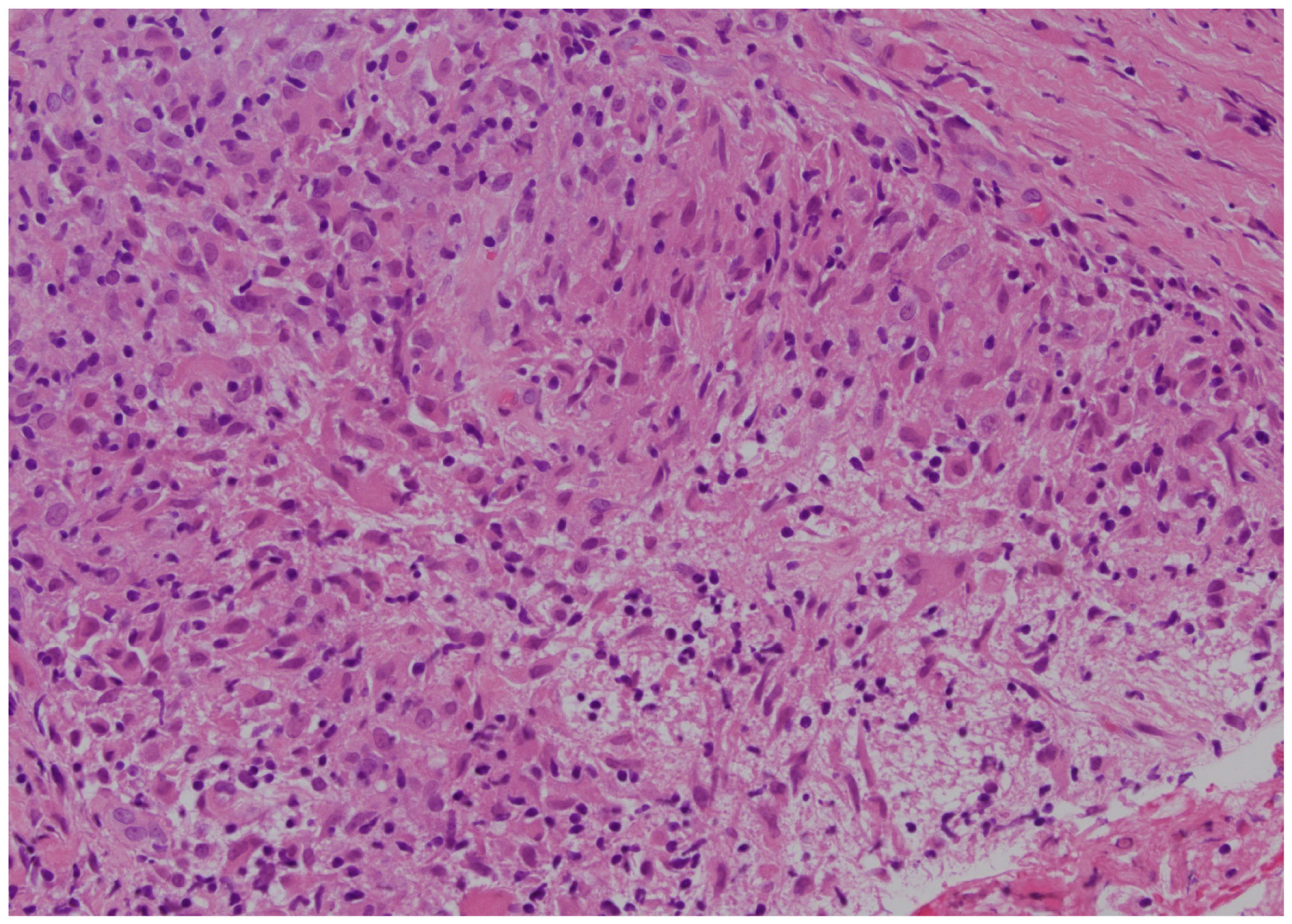
Histopathology of the biopsy specimen showing a granuloma formation.

A 22‐year‐old Vietnamese woman presented with a 2‐month history of anterior chest swelling and no underlying disease. Physical examination revealed swelling and tenderness around the anterior manubrium that worsened with deep breathing. Computed tomography (CT) revealed an osteolytic lesion in the manubrium, whereas magnetic resonance imaging showed an extra‐osseous expansion (Figure 1). A CT‐guided needle biopsy revealed granuloma formation (Figure 2), suggesting sarcoidosis, brucellosis, autoimmune vasculitis, or mycobacterial infection. A 3‐week mycobacterial culture indicated a *Mycobacterium tuberculosis* infection. The final diagnosis was manubrium tuberculosis.

Skeletal tuberculosis accounts for approximately 6%–10% of all extra‐pulmonary tuberculosis, equivalent to 1% of all cases.^1^ African and East Asian immigrants are at highest risk of skeletal tuberculosis.^1^, ^2^ However, manubrium/sternum involvement of tuberculosis is extremely rare, accounting for 1% of all skeletal tuberculosis cases.^1^, ^3^, ^4^ Risk factors for manubrium/sternal tuberculosis include residing in an endemic area, poor access to health care, immune suppression, previous sternotomy, diabetes mellitus, alcoholism, advanced age, and nosocomial exposure.^1^ As the great predecessors say, the present case reminds us that tuberculosis is a ubiquitous disease that can affect any part of the body. In this case, manubrium/sternal tuberculosis was cured after 9 months of antituberculosis treatment.

## AUTHOR CONTRIBUTIONS

**Tomohiro Fujiwara:** Conceptualization; writing – original draft. **Hiroyuki Yanai:** Investigation; writing – review and editing. **Hideharu Hagiya:** Conceptualization; writing – original draft.

## CONFLICT OF INTEREST STATEMENT

The authors declare no conflicts of interest.

## FUNDING STATEMENT

This study was supported by JSPS KAKENHI Grant Number 21K16709, 22K07303, and 22H03202.

## CONSENT

Informed consent for publication was obtained from the patient.

## ETHICAL APPROVAL

The manuscript was written following the COPE guidelines.

## Data Availability

Data sharing is not applicable to this article as no datasets were generated or analyzed.
